# Biosynthesis of Silver Nanoparticles Utilizing Leaf Extract of *Trigonella foenum-graecum* L. for Catalytic Dyes Degradation and Colorimetric Sensing of Fe^3+^/Hg^2+^

**DOI:** 10.3390/molecules28030951

**Published:** 2023-01-18

**Authors:** Monika Moond, Sushila Singh, Seema Sangwan, Parvesh Devi, Anuradha Beniwal, Jyoti Rani, Anita Kumari, Savita Rani

**Affiliations:** 1 Department of Chemistry, CCS Haryana Agricultural University, Hisar 125004, Haryana, India; 2 Department of Microbiology, CCS Haryana Agricultural University, Hisar 125004, Haryana, India; 3 Department of Plant Physiology, CCS Haryana Agricultural University, Hisar 125004, Haryana, India; 4 Department of Horticulture, CCS Haryana Agricultural University, Hisar 125004, Haryana, India

**Keywords:** silver nanoparticles, phytochemicals, catalytic degradation, bio-based sensors, green technology

## Abstract

The aqueous *Trigonella foenum-graecum* L. leaf extract belonging to variety HM 444 was used as reducing agent for silver nanoparticles (AgNPs) synthesis. UV–Visible spectroscopy, Particle size analyser (PSA), Field emission scanning electron microscopy coupled to energy dispersive X-ray spectroscopy (FESEM-EDX) and High-resolution transmission electron microscopy (HRTEM) were used to characterize AgNPs. Selected area electron diffraction (SAED) confirmed the formation of metallic Ag. Fourier Transform Infrared Spectroscopy (FTIR) was done to find out the possible phytochemicals responsible for stabilization and capping of the AgNPs. The produced AgNPs had an average particle size of 21 nm, were spherical in shape, and monodispersed. It showed catalytic degradation of Methylene blue (96.57%, 0.1665 ± 0.03 min^−1^), Methyl orange (71.45%, 0.1054 ± 0.002 min^−1^), and Rhodamine B (92.72%, 0.2004 ± 0.01 min^−1^). The produced AgNPs were excellent solid bio-based sensors because they were very sensitive to Hg^2+^ and Fe^3+^ metal ions with a detection limit of 11.17 µM and 195.24 µM, respectively. From the results obtained, it was suggested that aqueous leaf extract demonstrated a versatile and cost-effective method and should be utilized in future as green technology for the fabrication of nanoparticles.

## 1. Introduction

Recent developments in nanotechnology are linked to the creation of green technology for the synthesis of noble metal nanoparticles with controlled shape and size, which has unique properties that are very different from those of the corresponding bulk substances. Silver nanoparticles (AgNPs) are of particular interest due to their technological significance in numerous domains such as catalysis [1], optoelectronics [2], surface enhanced Raman spectroscopy [3], biomedicine [4], bio-sensors [5], and so forth. AgNPs can be made using a variety of physical and chemical methods, although these methods have unforeseen consequences such as environmental contamination, high energy usage, the emission of toxic and harmful chemicals, the usage of complicated equipment and the synthesis conditions. It is always preferable to adopt the green chemistry approach as an alternate source to conventional methods due to increasing awareness of the environmental impact of synthetic methods. As a result, a great deal of biological materials, including bacteria, algae, plants, and fungus have been found to generate AgNPs on their own without the use of additional reducing and stabilising agents [6]. The plant-mediated AgNPs production is superior to the technique utilizing microbes as it is less toxic to organisms, easily modified, and does not require maintaining cell culture [7]. Although ambient conditions can be used for plant-mediated biosynthesis, the time required for nanosynthesis is substantially longer than that for chemical methods. To mitigate this problem, microwave-assisted biosynthesis is used. Microwave irradiation can speed up the reaction. A practical way for the quick and simple green synthesis of silver nanoparticles uses plant extracts as both reducing and capping agents during microwave-assisted synthesis. It has a number of attractive features, including a faster reaction time, less energy use, and a higher product yield. Nanoparticles can develop and grow under uniform conditions because microwave irradiation rapidly and uniformly heats the reaction media [8]. Abboud et al. synthesized AgNPs using aqueous onion extract (*Allium cepa*) under microwave irradiation and reported that the usage of the microwave heating method is particularly crucial because it increases reaction kinetics, speeds up initial heating, and, as a result, improves reaction rates that lead to clean reaction products with quick consumption of raw materials and higher yields [9]. Josheph and Maethew reported microwave-assisted synthesis of silver nanoparticles using the leaf extract of *Aerva lanata* and studied the catalytic degradation of methyl orange and methylene blue dyes. They observed that with increase in concentration of catalyst, reaction time had decreased [10].

The annual herb *Trigonella foenum-graecum* L. (Fabaceae) is cultivated worldwide as a semi-arid crop. It is commonly known as Fenugreek and used as both spice and medicinal plant. Fenugreek is used to treat many ailments due to presence of various bioactive compounds, like apigenin, luteolin, orientin, quercetin, vitexin, isovitexin, saponins, amino acids, phenols, alkaloids, etc. [11]. These phytochemicals can be used to produce biogenic nanoparticles by acting as reducing and capping agents [12].

Nowadays, primary pollutants, like organic dyes, are the main contributor to water contamination. Numerous industries, including those in the textile, paper, culinary, pharmaceutical, cosmetic, leather, and printing, employ organic dyes extensively as colourants [13,14]. Scientists have recently become interested in the catalytic reduction of organic dyes and colorimetric sensing of heavy metal ions utilising biologically produced nanoparticles. Dyes and heavy metals are responsible for the water’s toxicity, which sicken people and animals severely. Therefore, it is desirable for a healthy existence that heavy metals and dyes should be removed specifically from drinking water [15].

Samari et al. synthesized AgNPs using mango leaf extract and reported catalytic reduction of MB and RhB dyes in 12 min and 8 min respectively. These AgNPs were used in colorimetric detection of Hg^2+^ [16]. Sooraj et al. reported green synthesis of silver nanoparticles (AgNPs) using the leaf extract of *Sida retusa*, and silver nanoparticles-catalyzed NaBH_4_ reduction reactions of methylene blue (MB) and methyl orange (MO) were studied, which showed excellent catalytic activities of nanoparticles. The reduction of MB was completed within 10 min with a constant (k) = 0.3761 ± 0.004 min^−1^ while reduction of MO occurred within 9 min with k = 0.2502 ± 0.003 min^−1^ [17]. Das et al. synthesized halloysite nanotubes-supported AgNPs composites using bio-inspired in situ oxidative polymerization of dopamine for highly efficient and catalytic degradation of methylene blue. The reduction of MB was completed within 10 min with rate 0.0013 s^−1^ [18]. Jyoti and Singh synthesized AgNPs using *Zanthoxylum armatum* leaves in degradation of hazardous dyes like Safranine O, Methyl red, Methyl orange, and Methylene blue. Due to their extremely high surface area and accelerated migration rate of electrons/holes to the surface of nanoparticles, AgNPs act as catalysts to enhance the efficiency of degradation [19]. Kadam et al. biosynthesized AgNPs using cauliflower (*Brassica oleracea var. botrytis*) waste extract and further tested their potential applications in photocatalytic degradation of methylene blue (MB) dye and Hg^2+^ biosensing. They reported that maximum MB dye degradation percentage of 97.57% was obtained within 150 min and biosensing studies showed that AgNPs were specifically able to detect up to 0.1 mg/l Hg^2+^ ions [20]. Das et al. synthesized cerium oxide nanotubes decorated silver nanoparticles (AgNPs) through in-situ polymerization of mussel inspired epinephrine chemistry and the efficiency of the nanocatalyst was assessed by the catalytic reduction of methylene blue (MB). They reported that MB dye degradation percentage of 94.01% was obtained within 7 min [21].

Although it is common to synthesize nanoparticles from plants, there has only been a limited amount of research on employing silver nanoparticles to clean dye effluent and sensing of metal ions. As a result, this field definitely needs greater assessment and evaluation. However, until now, research on the environment friendly synthesis of AgNPs using *Trigonella foenum-graecum* L. variety HM 444 and analysis of their potential environmental applications has not been thoroughly investigated. The aim of work presented here was to synthesize AgNPs using eco-friendly methods. The synthesized AgNPs were characterized using UV–Vis spectroscopy, PSA (Particle size analyser), FESEM-EDX (Field emission scanning electron microscopy coupled to energy dispersive X-ray spectroscopy), HRTEM (High resolution transmission electron microscopy), Selected area electron diffraction (SAED), and FTIR (Fourier Transform Infrared Spectroscopy). Further, the synthesized AgNPs have also been used in catalytic degradation of dyes and colorimetric sensing of heavy metal ions.

## 2. Results and Discussions

### 2.1. Synthesis of AgNPs

**Figure 1:**
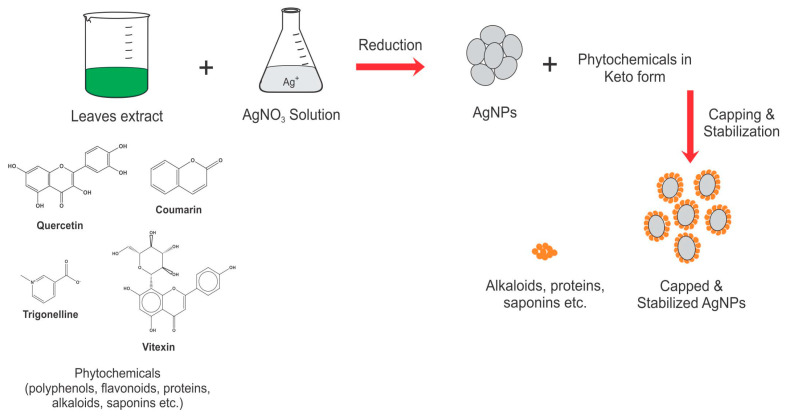
Probable mechanism for the synthesis of AgNPs.

*Trigonella foenum-graecum* L. leaves are rich source of numerous secondary metabolites of biological significance that can be used to produce biogenic nanoparticles as reducing and capping agents. Therefore, a green synthetic approach was employed to produce AgNPs by dissolving a 0.5 mL:25 mL (*v*/*v*) ratio of leaves extract and 1 mM Silver nitrate solution, respectively. A colour change from pale yellow to dark brown indicated the formation of AgNPs, which was confirmed by UV–Vis spectroscopic analysis. The findings were consistent with other publications in which different plant extracts were used to produce AgNPs and, after extract addition, the colour of aqueous silver nitrate solution (1 mM) changed from transparent to brown. The possible mechanism for synthesizing AgNPs utilising plant leaf extract was shown in Figure 1. Following the redox process, the presence of polyphenols and flavonoids in the leaf extract may contribute to the reduction of the silver ions to metallic form. Additionally, other secondary metabolites, including alkaloids, terpenoids, and saponins that are present in the leaf extract, may easily cap the produced Ag^0^ to stabilise it before converting it into silver nanoparticles (AgNPs).

### 2.2. Characterization of AgNPs

**Figure 2:**
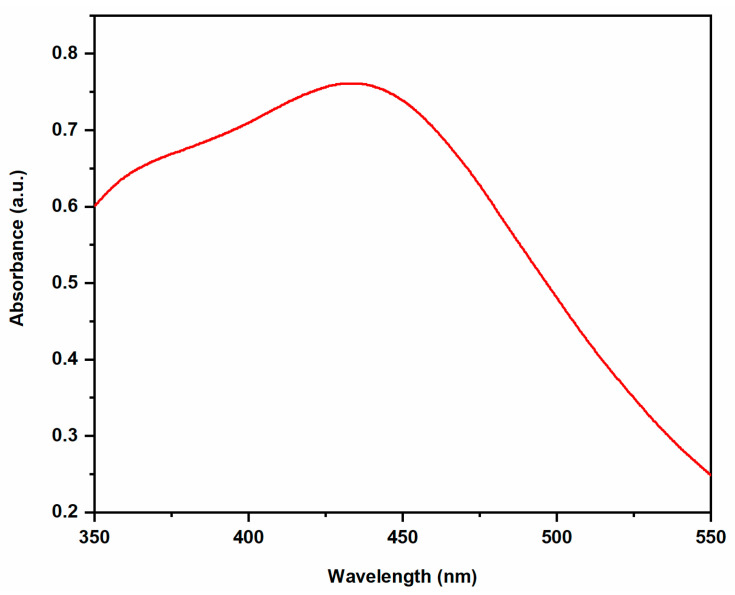
UV–Vis absorption spectrum of the biosynthesized AgNPs.

**Figure 3:**
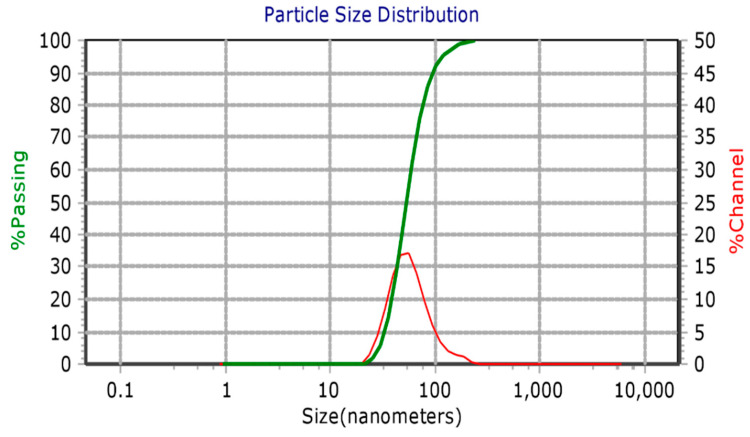
PSA of biosynthesized AgNPs.

UV–visible spectroscopy is an important technique to determine the formation and stabilization of AgNPs (Figure 2). UV-Visible analysis of biosynthesized AgNPs showed that an absorption peak at 448 nm as a result of the surface plasmon resonance (SPR) phenomenon [20]. The obtained results were consistent with previously reported literature, where *Salvadora persica* plant extract reduced silver ions to silver nanoparticles, and UV–visible absorption showed the SPR band for AgNPs to be in wavelength range of 350–550 nm [22]. The average particle size, polydispersity index (PDI) and zeta potential of biosynthesized AgNPs were found to be 60.15 nm, 0.2615, and −31.8 mV (Figure 3), respectively using a Particle Size Analyser (PSA). It actually measured the hydrodynamic diameter of the synthesized AgNPs and this diameter is not only related to the metallic core of the nanoparticles, as it is in the case of microscopic techniques, but the components present in the colloid and on the surface of the nanoparticles determine the thickness of the electrical double layer and its influence on the measured size of the particles [23].

**Figure 4:**
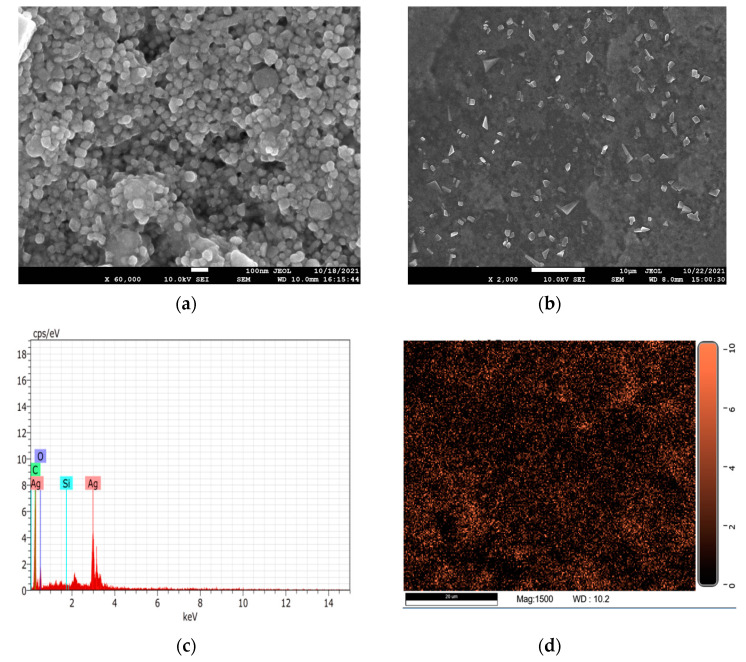
FESEM-EDX micrographic images (**a**) 100 nm scale; (**b**) 10 µm scale; (**c**) elemental mapping of AgNPs; (**d**) red color indicating Ag element.

The surface morphology, size, and chemical composition of the biosynthesized AgNPs were studied by FESEM-EDX analysis. The size of AgNPs was found in the range of 15–25 nm with an average diameter of 21 nm. Ag was computed and found to be 53.33% of the total weight in the EDX, while carbon, oxygen, and silicon weights were reported as being 27.65%, 18.93%, and 0.1%, respectively (Table 1). The Element Silicon comes from silicon carrier in FESEM detection, and signal of carbon and oxygen may be attributed to the proteins and carbohydrates capped on AgNPs. The chemical identity of the biosynthesized AgNPs, which were primarily made of Ag as evidenced by the red coloured dots in Figure 4, was supported by the elemental mapping of FESEM micrographs.

**Figure 5:**
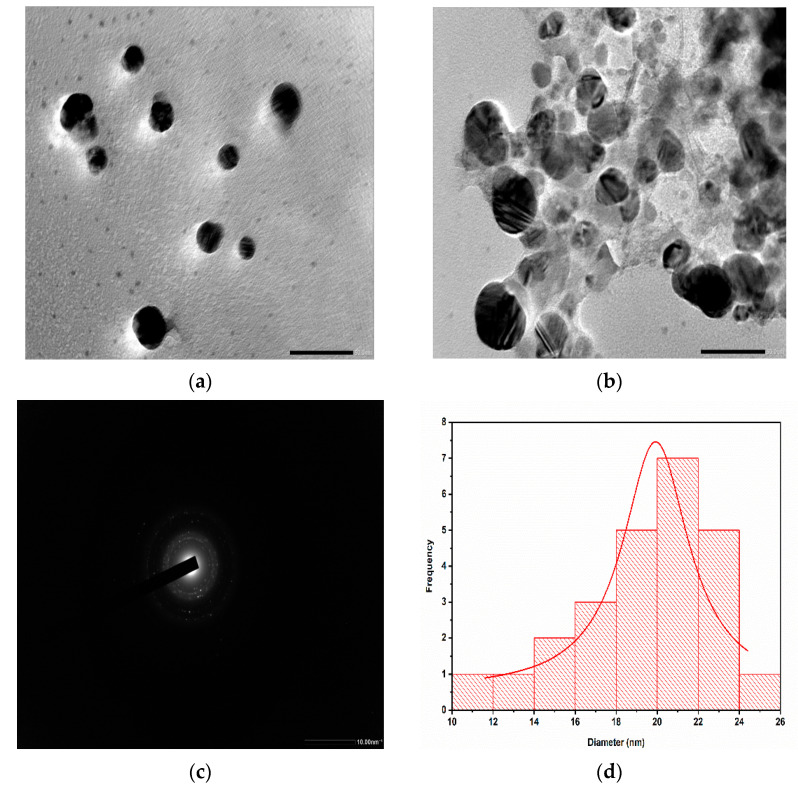
(**a**,**b**) HRTEM images of AgNPs in diverse magnification at (**a**) 20 nm scale (**b**) 50 nm scale (**c**) SAED pattern at 10 nm^−1^ (**d**) the particle histogram.

The HRTEM monographs in Figure 5a–d clearly showed the distribution of spherical AgNPs synthesized using *Trigonella foenum-graecum* L. leaves. The prepared nanoparticles had an uniform size distribution and were almost spherical in shape. The average particle size was 21 nm. The analysis also showed aggregations of nanoparticles and physical interaction, which might be attributed to biomolecules. The selected area electron diffraction (SAED) pattern with brilliant circular spot represented the crystalline nature of AgNPs.

**Figure 6:**
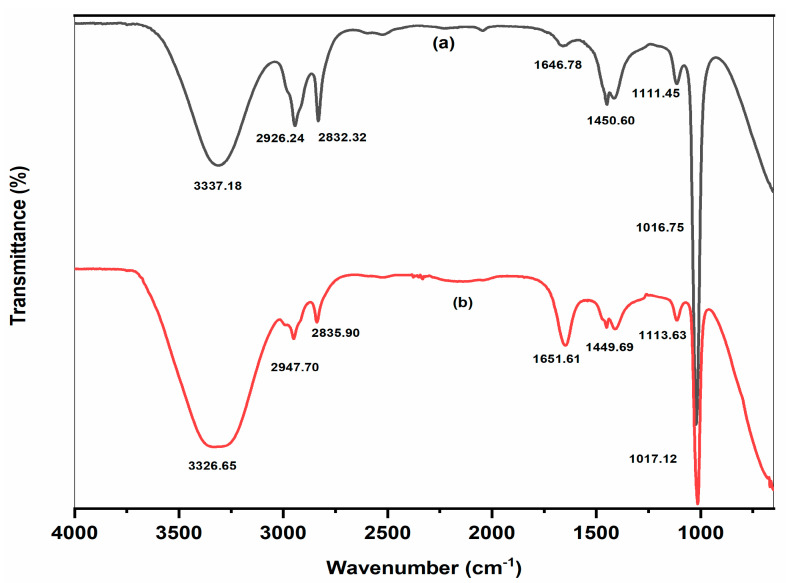
Comparative FTIR spectra of Trigonella *foenum-graecum* L. leaves extract (**a**) and biosynthesized AgNPs (**b**).

FTIR analysis of both plant leaf extract and AgNPs was carried out further, and their spectra were shown in Figure 6. According to FTIR data presented in Table 2, the leaves extract contained a variety of phytochemicals, including amino acids, amides, alkaloids, carbonyl, tannins, flavonoids, and phenolic hydroxyl with aromatic hydrocarbon. These biomolecules might contribute to the reduction of silver nitrate to AgNPs, which were stabilized by hydrogen bonding and the vander Waals electrostatic forces. High similarity in peaks position between extract and biosynthesized AgNPs revealed the presence of plant phytochemicals on the surface of biosynthesized AgNPs [24]. Hence, the successful fabrication of our desired silver nanoparticles was thus confirmed by all of the characterization results.

### 2.3. Catalytic Degradation of Dyes

#### 2.3.1. Degradation of Methylene Blue

**Figure 7:**
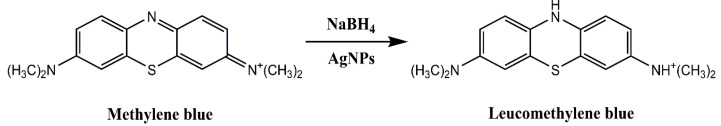
Schematic representation of reduction of Methylene Blue (MB).

**Figure 8:**
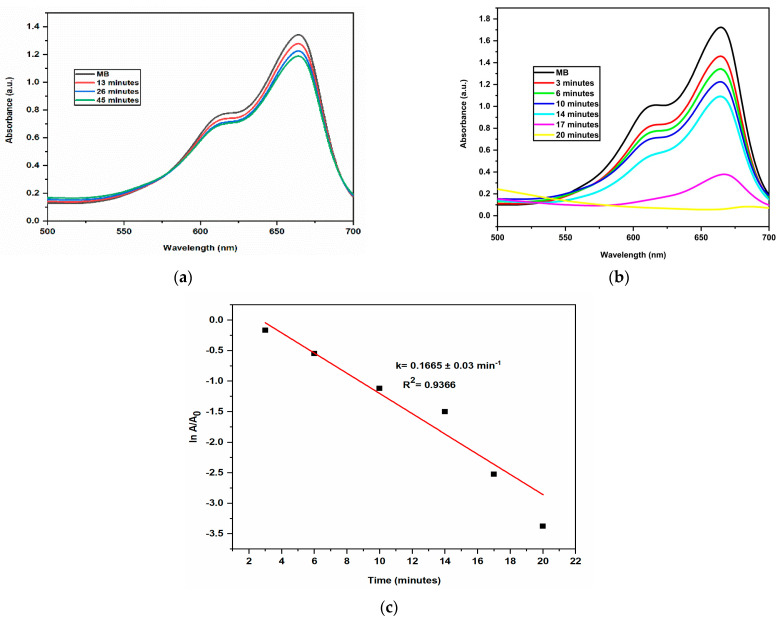
At 25 °C, successive UV–Vis absorbance spectra showing reduction of MB by NaBH_4_ (**a**) reaction mixture without AgNPs (**b**) reaction mixture containing 0.5 mL of AgNPs (**c**) Smooth plots of ln A/A_0_ versus time for the degradation of MB at 25 °C.

**Figure 9:**
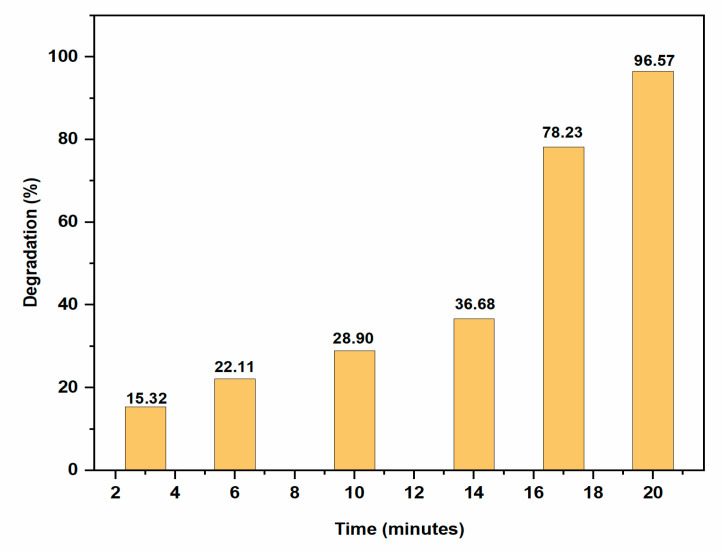
Bar graph of MB degradation at different time intervals.

Methylene Blue (MB) is a heterocyclic azo dye which is frequently discharged into the environment by the textile industry. It reduces oxygen levels on water’s surface, which has an adverse impact on aquatic animals and plant life [25]. It is recognised to be hazardous to humans in addition to being a risk to the environment. AgNPs of the appropriate size and shape have a high surface area to volume ratio, making them effective catalyst in dye degradation [26,27]. The extent of degradation of Methylene blue using AgNPs as catalyst was monitored by UV–visible spectrophotometer. Methylene blue dye’s absorption peak in water was found to be centred at 664 nm in the visible region [28,29]. AgNPs were added to the reaction mixture as a potential intermediate between MB dye and BH_4_^−^ ions. It first lowered the bond dissociation energy and improved the efficiency of the electron transfer between them and gradually the blue coloured MB solution changes to a colorless solution (leuco form) shown in Figure 7. The rate at which MB was reduced by NaBH_4_ increased in presence of AgNPs. In the presence of AgNPs, the absorption intensity diminished steadily over time and reached a minimum within 20 min. The reaction rate constant k for dye degradation by AgNPs was estimated with ln(A/A_0_) = −kt, as shown in Figure 8, indicating that the dye degradation followed pseudo-first-order kinetics [30]. The k value was reported as 0.1665 ± 0.03 min^−1^. The percentage of dye degradation was about 96.57 % within 20 min (Figure 9).

#### 2.3.2. Degradation of Methyl Orange

**Figure 10:**
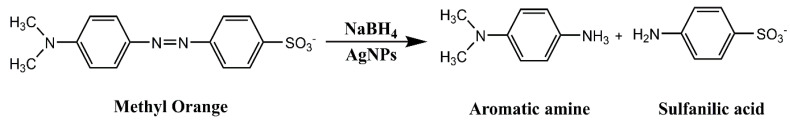
Schematic representation of reduction of Methyl Orange (MO).

**Figure 11:**
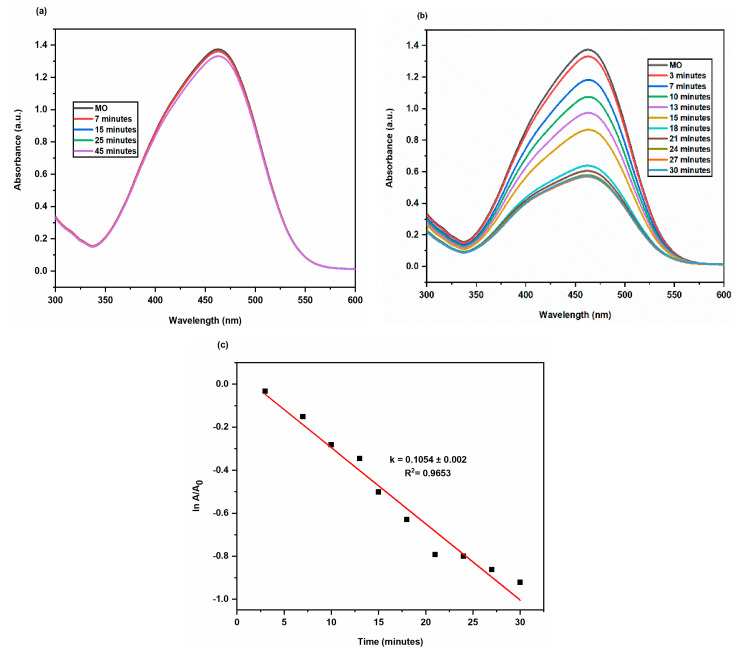
At 25 °C, successive UV–Vis absorbance spectra showing reduction of MO by NaBH_4_ (**a**) reaction mixture without AgNPs (**b**) reaction mixture containing 100 μL of AgNPs (**c**) Smooth plots of ln A/A_0_ versus time for the degradation of MO at 25 °C.

**Figure 12:**
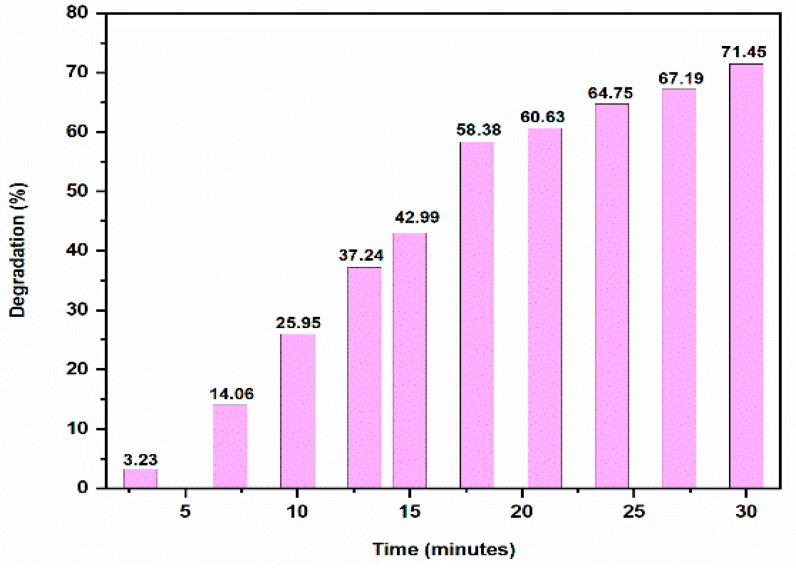
Bar graph of MO degradation at different time intervals.

A hazardous textile dye, Methyl Orange (MO) has terrible consequences on aquatic life. This azo dye exhibits an absorption peak of 464 nm in the UV-Vis absorption spectrum because of the N = N functional group [31]. The degradation of MO could not be brought about by the reducing agent NaBH_4_ alone as there was huge difference in redox potential of dye and NaBH_4_ and therefore, preventing the electron transfers between them. In contrast, MO degraded in the presence of AgNPs as a result of the electron relay effect. Since sodium borohydride and dyes have intermediate reduction potentials, AgNPs provide very simple electron transport between them [32]. The huge surface area of AgNPs increased the rate of deterioration (Figure 10). In the presence of AgNPs, the absorption intensity diminished steadily over time and reached a minimum within 30 min. The reaction rate constant k for dye degradation by AgNPs was estimated with ln(A/A_0_) = -kt, as shown in Figure 11, indicating that the dye degradation followed pseudo first order kinetics. The k value was 0.1054 ± 0.002 min^−1^. The percentage of dye degradation was about 71.45 % within 30 min (Figure 12).

#### 2.3.3. Degradation of Rhodamine B

**Figure 13:**
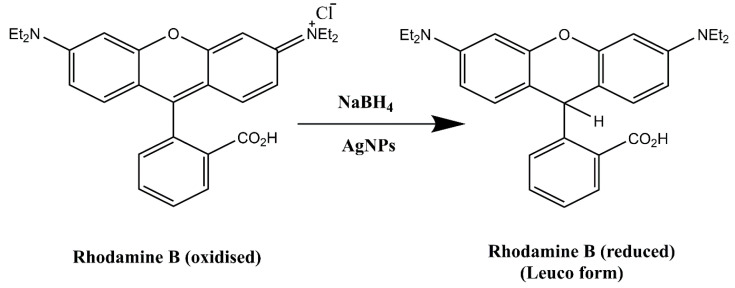
Schematic representation of reduction of Rhodamine B (RhB).

**Figure 14:**
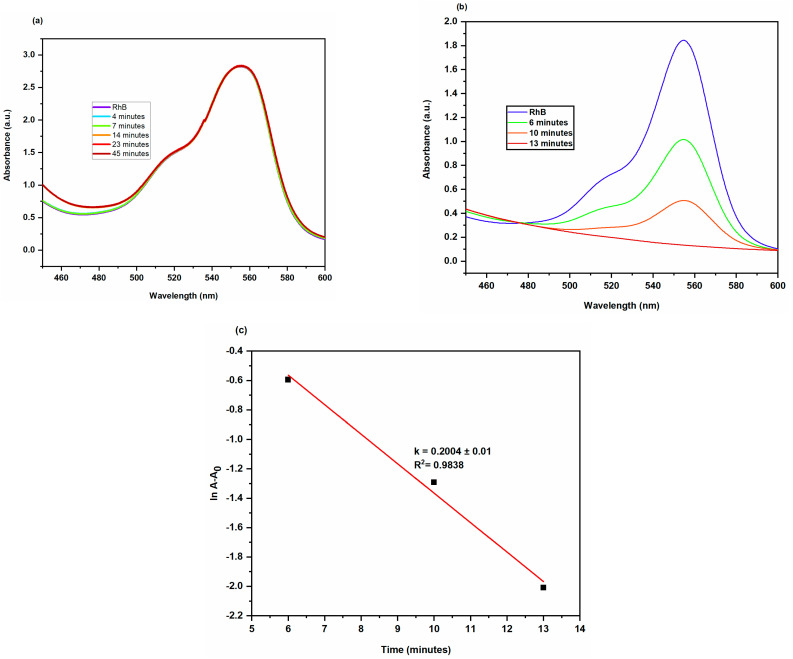
At 25 °C, successive UV-Vis absorbance spectra showing reduction of RhB by NaBH_4_ (**a**) reaction mixture without AgNPs (**b**) reaction mixture containing 0.2 mL of AgNPs (**c**) Smooth plots of ln A/A_0_ versus time for the degradation of RhB at 25 °C.

**Figure 15:**
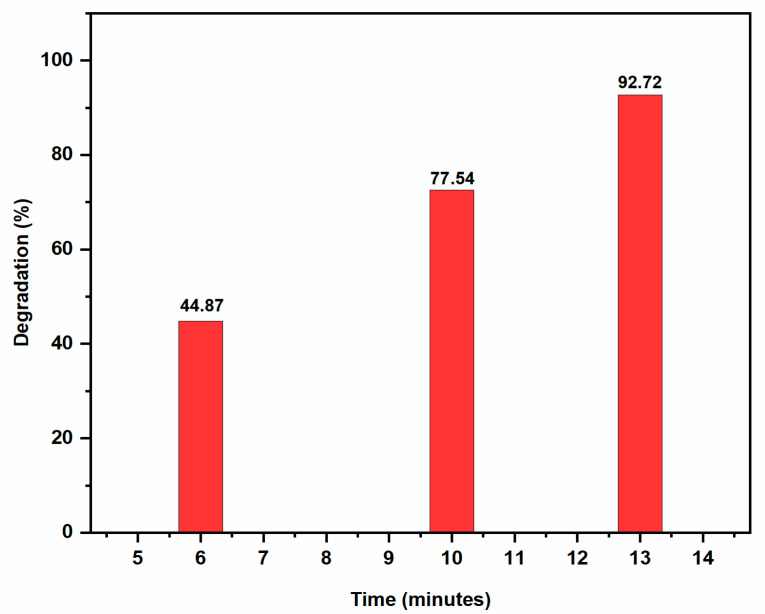
Bar graph of RhB degradation at different time intervals.

Rhodamine B (RhB) is a fluorescent dye from the xanthene family and commonly used in the pharmaceutical, paper, and cosmetic industries. Human eyes and skin are adversely affected by this dye pollutant [33]. Although the absorbance of the dye reduces just slightly in the absence of the catalyst (AgNPs), suggesting a relatively slow reaction rate, it abruptly decreases in the presence of the catalyst, indicating a faster reaction rate. AgNPs served as electron relays, moving electrons from the donor NaBH_4_ to the acceptor RhB dye because their redox potential was between that of NaBH_4_ (1.33 V) and RhB (0.48 V). By the exchange of electrons between BH_4_^−^ and the dye moiety on the surface of AgNPs, RhB was converted to leuco RhB which induced the dye degradation shown in Figure 13. The aqueous solution of RhB has a pink colour and its UV-Vis absorption spectrum shows high absorption between 200 and 700 nm, with a maximum at 554 nm. It is possible to kinetically follow the RhB degradation reaction by measuring the absorbance value of the peak at 554 nm as a function of time [34]. The reduction reaction of RhB by NaBH_4_ with AgNPs catalyst at a temperature of 25 °C was depicted in Figure 14 as the UV-Vis absorption spectra. The intensity of the absorption diminished when AgNPs were present. The absorption intensity diminished steadily over time and reached a minimum within 13 min. The degradation of dye fitted well with pseudo first order equation and the graph generated by plotting ln A/A_0_ versus time showed linear behaviour [35]. AgNPs had catalytic rate constant (k) of 0.2004 ± 0.01 min^−1^. The percentage of dye degradation was about 92.72% within 13 min (Figure 15).

Awad et al. synthesized AgNPs *Trigonella foenum graecum* L. seed extract and the photocatalysis of AgNPs to induce RhB degradation was studied. It was observed that the photodegradation of RhB dye was increased by passing time and the photodegradation nearly (93%) was achieved after 216 h. The efficient adsorption between AgNPs and RhB molecules may also be encouraged by the layer of reducing agent on the surface of AgNPs. As a result, for smaller particles, the oxidation reduction reaction between the active RhB and reducing agent can happen more quickly, effectively, and easily. The outcomes showed that AgNPs’ strong reactivity and substantial surface area served as an effective photocatalyst for dye degradation under UV light, confirming their superior ability to reduce and eliminate dye pollution [36]. A comparative study of catalytic degradation of all three dyes using AgNPs was given in Table 3.

#### 2.3.4. Colorimetric Sensing of Metal Ions

**Figure 16:**
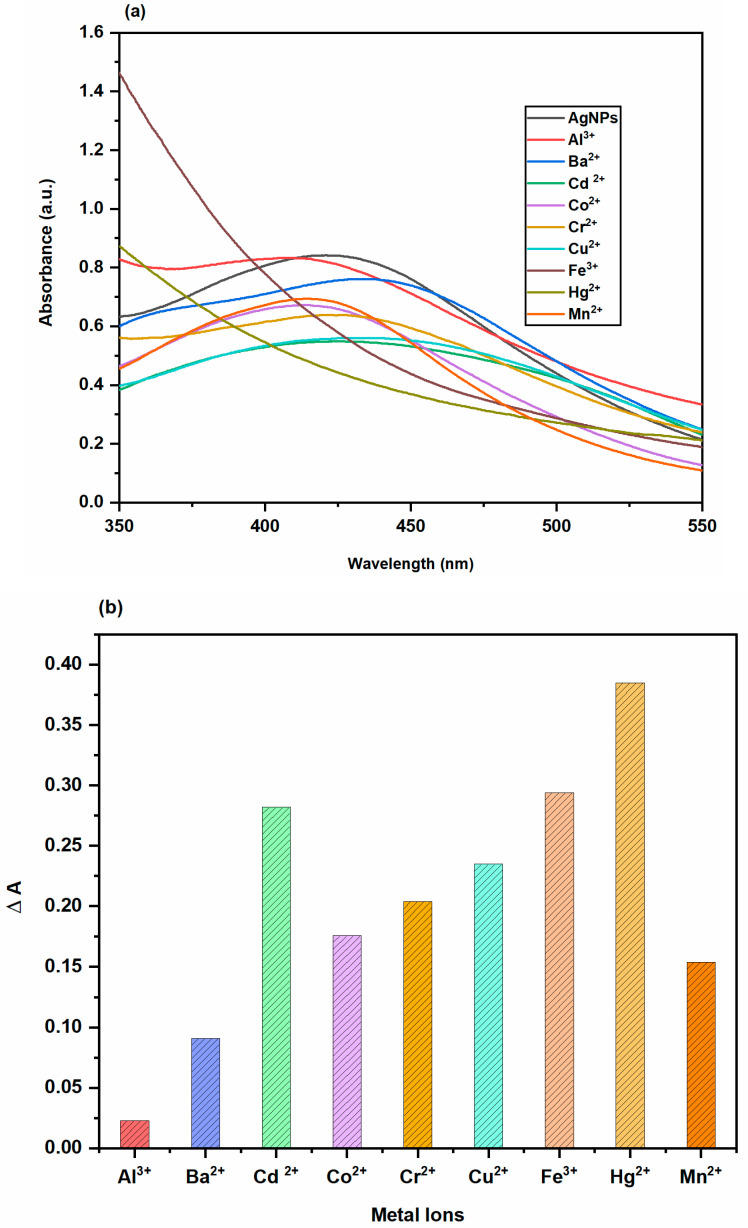
(**a**) UV-Vis spectra of AgNPs solutions in the absence and presence of various metal ions (**b**) The changes in λ_SPR_ values of AgNPs solution containing various metal ions (ΔA = A_0_ − A).

The colorimetric detection of metal ions using AgNPs made from *Trigonella foenum-graecum* L. leaves was explored in this study. First, it had been determined whether the biosynthesized AgNPs were selective for a range of metal ions (Ba^2+^, Al^3+^, Mn^2+^, Co^2+^, Cu^2+^, Cr^2+^, Cd^2+^, Hg^2+^ and Fe^3+^) when used as a colorimetric sensor. The biosynthesized AgNPs solution was mixed with uniform concentrations of various metal salt solutions (1000 μM) to achieve this. The generated solutions’ colour changes was then first visually examined, and after that, the absorption spectra of AgNPs solutions in the presence of various metal ions were recorded. AgNPs solutions’ UV-Vis spectra were displayed in Figure 16 along with a bar chart that was made utilising the variations in the solution’s absorbance in the absence and presence of various metal ions. Figure 16a showed that the colour of AgNPs solutions containing Hg^2+^ and Fe^3+^ ions changed from brown to colourless and that the SPR of these solutions vanished, whereas the colours and SPR intensities of AgNPs solutions containing Ba^2+^, Al^3+^, Mn^2+^, Co^2+^, Cu^2+^, Cr^2+^ and Cd^2+^ showed very small or even no significant changes. The bar of AgNPs solution in the presence of Hg^2+^ and Fe^3+^ ions was also substantially higher than that in the presence of the other metal cations, as shown in Figure 16b, indicating that the other metal cations had minimal effect on the biosynthesized AgNPs. The results obtained demonstrated unequivocally that the biosynthesized AgNPs were highly selective for Hg^2+^ and Fe^3+^ ions when compared to other cations. The biosynthesized AgNPs’ ability to detect Hg^2+^ and Fe^3+^ ions was based on the reduction oxidation reaction. The redox reactions that occur spontaneously are those with a positive cell potential (E^0^_cell_). AgNPs and Hg^2+^ ions undergo a spontaneous redox reaction [37].

(1) $$2Ag+{2Hg}^{2+}\to 2Ag+{Hg}_{2}^{2+}\;{E^{0}}_{\mathrm{cell}}=+0.12V$$

Unlike Hg^2+^ ions, Ferric ions (Fe^3+^) lack the ability to oxidise elemental silver (AgNPs) due to negative E^0^_cell_ values. The presence of halide ions in the detecting medium (Cl^−^ ions derived from FeCl_3_ metal salt solution) significantly reduced the reduction potential of the silver species by strongly coordinating with silver species. As the potential of Ag^+^/Ag was reduced, allowing elemental silver to be oxidised by ferric ions by producing a positive E^0^_cell_ value. As a result, the redox interaction between Fe^3+^ ions and AgNPs occured spontaneously [38,39].

(2) $$Ag+{Fe}^{3+}\to {Ag}^{+}+{Fe}^{2+}$$

**Figure 17:**
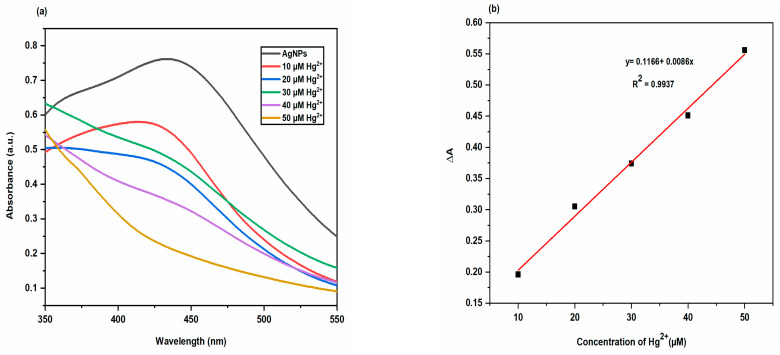
(**a**) UV-Vis spectra of AgNPs solutions in the absencs and presence of Hg^2+^ ions at different concentrations (**b**) Plot of ∆A values for biosynthesized AgNPs solutions containing Hg^2+^ ions at different concentrations versus Hg^2+^ concentration.

In order to ascertain the sensitivity of the biosynthesized AgNPs colorimetric sensor for Hg^2+^ sensing, the variations in the AgNPs solution colour and SPR intensity for various concentrations of Hg^2+^ were evaluated. The results were displayed in Figure 17. The colour of AgNPs solution changed progressively from brown to colourless as the concentration of Hg^2+^ increased from 10 µM to 50 µM, and the SPR intensities of AgNPs solutions containing Hg^2+^ ions at different concentrations decreased gradually (Figure 17a). These findings suggested that the biosynthesized AgNPs exhibited a colorimetric response that was Hg^2+^ concentration dependent [40,41]. The biosynthesized AgNPs colorimetric sensor’s limit of detection (LOD) for Hg^2+^ ions was calculated from three times the standard deviation of the blank signal (LOD = 3σ/s). AgNPs colorimetric sensor’s LOD for Hg^2+^ ions was found to be 11.17 µM. Karthiga and Anthony reported that AgNPs synthesized using different plant extracts (neem leaves and bark, mango leaves, tea and pepper seeds) demonstrated the ability to detect heavy metal ions in aqueous solution over a wide pH range. AgNPs synthesized using neem leaf and bark extracts, mango leaf, tea extracts and pepper seed extracts selectively detected Hg^2+^ ions [42]. Roy et al. evaluated the metal ion detecting ability of AgNPs using *Dahlia pinnata* leaf extract. They reported that with the addition of Cd^2+^, Cr^3+^, Zn^2+^ and Pb^2+^ metal ions into AgNPs solution, there was no specific color change except broadening and a small shift of the absorbance peaks was observed, but addition of Hg^2+^ ion into AgNPs solution instantly turned it colorless from light brown, and thus established the selective colorimetric detection of Hg^2+^ [43].

**Figure 18:**
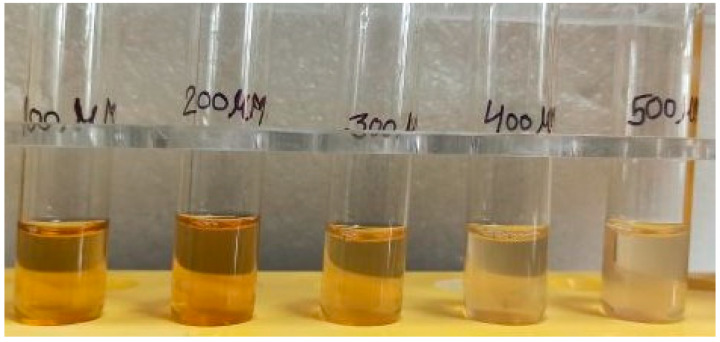
Image displaying reaction between Fe^3+^ ions (100 μM to 500 μM) as synthesized AgNPs.

**Figure 19:**
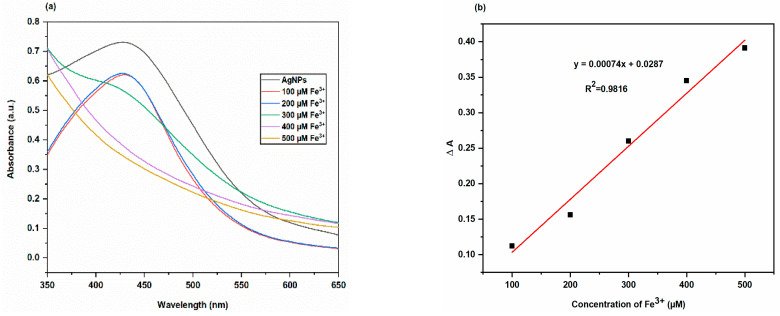
(**a**) UV-Vis spectra of AgNPs solutions in the absence and presence of Fe^3+^ ions at different concentrations (**b**) plot of ∆A values for biosynthesized AgNPs solutions containing Fe^3+^ ions at different concentrations versus Fe^3+^ concentration.

To ascertain the sensitivity of the biosynthesized AgNPs colorimetric sensor for Fe^3+^ sensing, the changes in the AgNPs solution colour and SPR intensity for various concentrations of Fe^3+^ were measured. The results were shown in Figure 18. It was clear that the colour of the AgNPs solution changed gradually from brown to colourless as the concentration of Fe^3+^ ions increased from 100 μM to 500 μM (Figure 18), and that the SPR intensities of AgNPs solutions containing Fe^3+^ ions at different concentrations gradually decreased (Figure 19). These findings suggested that the Fe^3+^ concentration dependent on colorimetric response of the biosynthesized AgNPs. The biosynthesized AgNPs colorimetric sensor’s limit of detection (LOD) for Fe^3+^ ions was calculated from three times the standard deviation of the blank signal [44]. AgNPs colorimetric sensor’s LOD for Fe^3+^ ions was found to be 195.24 µM. Uzunoğlu et al. synthesized AgNPs using orchid tree (*Bauhinia variegata*) leaf extract and carried out the colorimetric detection studies of the different species such as Na^+^, K^+^, Mg^2+^, Ba^2+^, Ni^2+^, Mn^2+^, Cu^2+^, Zn^2+^, Cd^2+^ and Fe^3+^ with the biosynthesized AgNPs. They reported that these biosynthesized AgNPs showed a strong surface plasmon resonance (SPR) around 430 nm. The maximum decrease in the SPR and disappearance of color was observed only in the presence of Fe^3+^. Thus, these AgNPs can be used for the sensitive and selective detection of Fe^3+^ ions in aqueous solution with a linear range of 6–100 μM and a detection limit of 2.08 × 10^−6^ M [45,46].

## 3. Materials and Methods

### 3.1. Chemicals and Collection of Plant Materials

All the chemicals used were of analytical grade. Silver nitrate (AgNO_3_), Sodium borohydride (NaBH_4_), Methylene Blue, Methyl Orange, and Rhodamine B dyes were purchased from Himedia Private Limited. All experiments used deionized water from a Milli-Q system. The leaves of *Trigonella foenum-graecum* L. belonging to variety Hisar Mukta (HM) 444 were cultivated in Research farms of Chaudhary Charan Singh Haryana Agricultural University, Hisar.

### 3.2. Preparation of Plant Extract

Fresh and healthy leaves of *Trigonella foenum-graecum* L. (HM 444) were washed well with deionised water. The leaves were dried and crushed into fine powder using grinder. 10 g of powdered leaves were heated with 100 mL of deionised water at 60 °C for 45 min. After that, the leaf extract was centrifuged for 15 min at 7500 rpm while being filtered through Whatman filter paper No. 1. Finally, the obtained leaves extract was stored at 4 °C for further experimental assays.

### 3.3. Synthesis of AgNPs

The leaves extract was used as a reducing and capping agent to convert silver salt to its metallic form. 0.5 mL extract was added to 25 mL of 1 mM AgNO_3_ solution. The synthesis procedure was carried out in a commercially available Discover LabmateTM microwave autoclave reactor provided by CEM GmbH (Kamp-Lintfort, Germany). A 50 mL glass high pressure vessel with a magnetic stirrer was part of this apparatus. The microwave synthesis of AgNPs was carried out at temperature 60 °C for 5 min. The stirrer speed (600 rpm) and power (420 W) were set during synthesizing procedure. The reaction mixture solution underwent a 15 min centrifugation process at 15,000 rpm to precipitate AgNPs, which were then dried at 37 °C and used for further analysis.

### 3.4. Characterization of AgNPs

The UV-Vis absorption spectrum of AgNPs in the wavelength range of 350–550 nm was recorded using a UV-Vis double beam spectrophotometer (Model UV 1900, Shimadzu, Kyoto, Japan). Deionised water was used as control. The hydrodynamic size distributions, zeta potential, and polydispersity index (PDI) of nanoparticles were determined by using Particle Size Analyzer (PSA Microtracnanotrac wave II, Anton Paar, Graz, Austria) instrument. The surface morphology of biosynthesized AgNPs was analyzed with the help of Field emission scanning electron microscopy (FESEM, JSM-7610FPlus, JEOL Ltd., Akishima, Japan) operating at an accelerating voltage of 0.1 to 30 kV equipped with an energy dispersive X-ray spectroscopy (EDX) detector for elemental mapping. On a JEM/2100 PLUS running at 200 kV, High resolution transmission electron microscopy (HRTEM) was successfully performed. A drop of the biosynthesized AgNPs dispersed in ethanol was placed at 400 mesh copper grid coated with a holey carbon film for the HRTEM investigations. The chemical composition of plant extract and AgNPs were studied by using FTIR spectrophotometer (Perkin Elmer, Waltham, MA, USA) and spectra was taken in the 4000–650 cm^−1^ region.

### 3.5. Catalytic Activity

Methylene Blue (MB), Methyl Orange (MO), and Rhodamine B (RhB) were reduced with sodium borohydride (NaBH_4_) in the presence of AgNPs in order to evaluate the effectiveness of the catalytic activity of the synthesised AgNPs. A freshly prepared mixture of 10 mL of 4 × 10^−5^ M MB and 0.5 mL of 0.05 M NaBH_4_ solution was vortexed for 5 min. Similar to this, 10 mL of freshly prepared 4 × 10^−5^ M MO was mixed with 100 μL of freshly prepared 0.05 M NaBH_4_ solution, and 0.2 mL of freshly prepared 0.05 M NaBH_4_ was added to 10 mL of RhB, and each was then stirred for 5 min. To all MB, MO, and RhB solutions, 0.5 mL, 100 μL, and 0.2 mL of biosynthesized AgNPs were added, respectively, and stirred vigorously for 2 min. The UV-Vis spectrum of the reaction mixture of MB was recorded for a period of 20 min at a wavelength range of 500–700 nm at 25 °C, while the absorption spectrum of MO was recorded for a period of 30 min at a wavelength range of 300–600 nm at 25 °C, and that of RhB was recorded for a period of 13 min at a wavelength range of 440–600 nm at 25 °C. The experiment was carried out in a 3.0 mL standard quartz cuvette. The dye absorbance of the resultant supernatant of the control and test solutions was measured at the appropriate wavelength. As a control, only dye solution of appropriate concentration was used. The following formula was used to determine the percentage of dye degradation in the test and control at the respective time interval:

(3) $$\mathrm{Degradation}(\%)=100\times (A_{0}-A)∕A_{0}$$

where ‘A_0_’ denotes the dye solution’s initial absorbance and ‘A’ denotes the dye solution’s absorbance as measured after each time interval.

### 3.6. Colorimetric Sensing

Through a series of experiments, the colorimetric detection studies of various species, including Ba^2+^, Al^3+^, Mn^2+^, Co^2+^, Cu^2+^, Cr^2+^, Cd^2+^, Hg^2+^ and Fe^3+^ with the biosynthesized AgNPs, were conducted. After thoroughly stirring the mixture containing 2.0 mL of AgNPs solution, 2.0 mL of 1000 μM metal ion solution was added. Utilizing a UV-Vis spectrophotometer, the absorbance value of the prepared solution was measured, and their spectra were recorded in the 350–550 nm wavelength range. AgNPs’ Surface Plasmon Resonance (SPR) decreased to its lowest possible level and their color completely vanished only when Hg^2+^ and Fe^3+^ were present. Therefore, by reducing the SPR intensity of AgNPs, different concentrations of Hg^2+^ (10–50 μM) and Fe^3+^ (100–500 μM) were used to calculate the limit of detection.

## 4. Conclusions

In this study, a successful, rapid, and green synthesis of AgNPs was achieved using *Trigonella foenum-graecum* L. leaves extract (HM 444) as an effective reducing and capping agent. Different methods, including like UV-Visible Spectroscopy, FESEM-EDX, HRTEM, SAED, and FTIR, were successfully used to characterize the synthesized AgNPs. The potential catalytic degradation of MB, MO, and RhB dyes were also demonstrated by AgNPs. In comparison to MO and RhB, the synthesized catalyst demonstrated greater degradation efficiency against MB. The AgNPs’ outstanding degradation of dyes points to its possible use in the treatment of industrial dye effluent and the purification of water. The biosynthesized AgNPs were subsequently tested as a colorimetric sensor and found to be a reliable colorimetric sensor for the detection of Hg^2+^ and Fe^3+^ ions in aqueous solution. Overall, these findings imply that the AgNPs synthesized in this study can serve as both a reliable colorimetric sensor and a strong catalytic degradation analyte. The selective colorimetric sensing of Hg^2+^ and Fe^3+^ ions by green synthesized AgNPs demonstrated the multi-functional utility of plant extracts in green nanotechnology and environmental sensor applications. This novel method for the environment-friendly synthesis of AgNPs has a number of attractive features and provides an effective and affordable means of protecting the environment. In future, researchers can use AgNPs for the purpose of wastewater purification through the removal of heavy metals and dyes by exploring their unique properties, such as high surface-to-volume ratio, tunable pore size, high sensitivity, reactivity, adsorption capacity, catalytic activity and ease of functionalization. Therefore, these plant mediated AgNPs can have potential application in the field of wastewater treatment.

## Figures and Tables

**Table 1:** EDX analysis parameters of AgNPs.

Element	Weight%	Atomic%
Carbon	27.65	57.79
Oxygen	18.93	29.71
Silver	53.33	12.41
Silicon	0.10	0.09

**Table 2:** FTIR analysis of *Trigonella foenum-graecum* L. leaves extract and biosynthesized AgNPs.

S.No.	FTIR Peaks (cm^−1^)	Assignment of Peaks	Range (cm^−1^)
1	3326.65, 3337.18	-OH or –NH stretching vibrations	3200–3500
2	2835.90, 2832.32, 2947.70, 2838.78, 2926.24	C-H stretching vibration of -CH_3_ groups, aldehydes H-C=O	2700–2950
3	1651.61, 1646.78	-C=O Stretching of amide groups, N–H of the proteins	1650–1850
4	1450.60, 1449.69	stretching vibration of alkenes C=C in aromatic rings	1380–1465
5	1113.63, 1111.45, 1113.78, 1017.12, 1016.75	-C-O-C or C-N stretching	1016–1250

**Table 3:** A Comparative study table for catalytic degradation of dyes using AgNPs.

Dyes (10 mL of 4 × 10^−5^ M)	Volume of AgNPs	Volume of 0.05 M NaBH_4_	Rate Constant (min^−1^)	R^2^	Degradation (%)	Reduction Time (min)
Methylene Blue (MB)	0.5 mL	0.5 mL	0.1665 ± 0.03	0.9366	96.57	20
Methyl Orange (MO)	100 μL	100 μL	0.1054 ± 0.002	0.9653	71.45	30
Rhodamine B (RhB)	0.2 mL	0.2 mL	0.2004 ± 0.01	0.9838	92.72	13

## Data Availability

Not applicable.
